# Epithelial plasticity enhances regeneration of committed taste receptor cells following nerve injury

**DOI:** 10.1038/s12276-022-00924-8

**Published:** 2023-01-11

**Authors:** Anish Ashok Adpaikar, Jong-Min Lee, Dong-Joon Lee, Hye-Yeon Cho, Hayato Ohshima, Seok Jun Moon, Han-Sung Jung

**Affiliations:** 1 Division in Anatomy and Developmental Biology, Department of Oral Biology, Taste Research Center, Oral Science Research Center, BK21 FOUR Project, Yonsei University College of Dentistry, Seoul, South Korea; 2 Division of Anatomy and Cell Biology of the Hard Tissue, Department of Tissue Regeneration and Reconstruction, Niigata University Graduate School of Medical and Dental Sciences, Niigata, Japan; 3 Department of Oral Biology, BK21 FOUR Project, Yonsei University College of Dentistry, Seoul, South Korea

**Keywords:** Reprogramming, Adult stem cells

## Abstract

Taste receptor cells are taste bud epithelial cells that are dependent upon the innervating nerve for continuous renewal and are maintained by resident tissue stem/progenitor cells. Transection of the innervating nerve causes degeneration of taste buds and taste receptor cells. However, a subset of the taste receptor cells is maintained without nerve contact after glossopharyngeal nerve transection in the circumvallate papilla in adult mice. Here, we revealed that injury caused by glossopharyngeal nerve transection triggers the remaining differentiated *K8*-positive taste receptor cells to dedifferentiate and acquire transient progenitor cell-like states during regeneration. Dedifferentiated taste receptor cells proliferate, express progenitor cell markers (K14, Sox2, PCNA) and form organoids in vitro. These data indicate that differentiated taste receptor cells can enter the cell cycle, acquire stemness, and participate in taste bud regeneration. We propose that dedifferentiated taste receptor cells in combination with stem/progenitor cells enhance the regeneration of taste buds following nerve injury.

## Taste: cellular processes for regenerating taste buds

Studies in mice reveal details of how taste receptor cells in the mouth can regenerate to restore the sense of taste after nerve injury, with implications for understanding and perhaps treating nerve damage affecting taste in humans. Taste receptor cells normally survive between 8 and 24 days and are continually renewed in a process requiring contact with a healthy nerve. Han-Sung Jung and colleagues, at Yonsei University, Seoul, South Korea, found that cutting the relevant nerves in mice caused some of the existing taste receptor cells to revert to an earlier stage in their development to participate in taste bud regeneration. These previously mature cells acquired some characteristics of stem cells, with the potential to develop into a variety of cell types. The researchers now plan to more fully investigate the molecular mechanisms involved.

## Introduction

The sense of taste is of fundamental importance, as it aids in the detection of stimuli to identify nutritious substances and to avoid toxic substances^1,2^. Despite the sensory nature of taste receptor cells, they are epithelial cells possessing a lifespan of 8–24 days and are renewed throughout life^3–5^. Leucine rich repeats containing G protein coupled receptor 5 (*Lgr5*)-positive cells are stem/progenitor cells, keratin 14 (*K14*) indicates progenitor cells, which give rise to taste and nontaste epithelial cells of the circumvallate papillae (CVP), and keratin 8 (*K8*) marks differentiated taste receptor cells^6–9^.

Injury models alter the cell identity and hence allow analysis of the cell behavior and dynamics of the cells during regeneration^10^. Transection of the gustatory nerve leads to degeneration of taste receptor cells at 2 weeks, and regenerated cells gradually appear after nerve contact is reestablished^11,12^. Previous studies have reported that not all taste receptor cells in the CVP degenerate after transection of the glossopharyngeal nerve^13,14^. However, the role and fate of the remaining taste receptor cells remain largely unknown.

Rapidly renewing epithelial tissues are capable of robust regeneration utilizing resident adult stem cells to replace lost differentiated cells both under homeostasis and during regeneration^15^. In recent studies, cellular plasticity has been recognized as a method of regeneration after injury in various tissues^16,17^. The intestine and colon are maintained by stem cells that express *Lgr5* during homeostasis. However, due to ablation of stem cells by injury, *Lgr5*-negative precursor cells that persist after injury were shown to acquire a stem cell-like state and regenerate the tissue via dedifferentiation^18,19^. However, it remains unknown whether cellular plasticity is acquired by taste receptor cells that persist after injury for the regeneration of taste buds. This study aimed to investigate the fate of the remaining taste receptor cells after glossopharyngeal nerve transection (GLx) in the CVP of mice.

We determined that a subset of taste receptor cells that were maintained in the circumvallate papilla after nerve injury formed taste and nontaste epithelium during regeneration. This finding indicated that the injury induced differentiated taste receptor cells acquire stem/progenitor-like characteristics to participate in the regeneration of the taste bud.

## Materials and methods

### Animals

All animal experiments were approved by the Yonsei University Health System Institutional Animal Care and Use Committee (YUHS-IACUC) in accordance with the Guide for the Care and Use of Laboratory Animals (National Research Council, USA). The animal study plan for these experiments (2019-0312) was reviewed and approved by this committee. All experiments were performed in accordance with the guidelines of this committee.

Mice were housed in a temperature-controlled room (22 °C) under artificial illumination (lights on from 05: 00 to 17: 00) and 55% relative humidity, and they had ad libitum access to food and water. All the operational procedures were performed under deep anesthesia. Adult mice (purchased from Koatech Co., Pyeongtaek, Korea) were housed in a temperature-controlled room (22 °C) under artificial lighting (lights on from 05: 00 to 17: 00) and 55% relative humidity with access to food and water ad libitum. The mice used in the study were adults (>6 weeks of age). For tracing the fate of stem cells, *Lgr5*^EGFP-IRES-creERT2^ ^20^, progenitor cell *K14*^CreERT/+^ ^21^, and differentiated taste receptor cell *K8*^CreERT2/+^ ^22^ mice were bred with *R26*^*flox-STOP-tdTomato*^ ^23^ mice to generate *Lgr5*^EGFP-CreERT2/+;^ *R26R*^Tom/+^, *K14*^CreERT/+;^ *R26R*^Tom/+^ and *K8*^CreERT2/+^; *R26R*^Tom/+^ mice.

### BrdU and IdU labeling

We injected 5-bromo-2-deoxyuridine (BrdU, B5002, Sigma, MO, USA) into mice two times a day for six consecutive days (100 mg/kg per injection) to label all the proliferating cells of the circumvallate papilla. The labeled mice were sacrificed at 12 h (no chase) to identify proliferating cells and 2 and 6 weeks (chase) from the last BrdU injection to identify label-retaining cells (LRCs) in the circumvallate papilla. For identification of the LRCs in the regenerated taste bud, the mice were injected with BrdU as described above. Bilateral GLx was performed on the labeled mice 4 weeks after the last injection of BrdU to identify only LRCs. The mice were then sacrificed at 2 and 6 weeks after GLx to identify the LRCs in the regenerated taste bud. Then, 5-iodo-2′-deoxyuridine (IdU, I7125, Sigma, MO, USA) was injected into the mice (100 mg/kg) 1 h before sacrifice at 4 weeks after GLx to identify proliferating LRCs in the taste bud.

### Glossopharyngeal Nerve Transection (GLx)

The adult mice were anesthetized by injection with anesthetic (Rumpun: Zoletil: saline = 1: 5: 6, 60∼70 μl/mouse), and all efforts were made to minimize suffering. GLx was performed based on the method described in^12^. In brief, an incision was made along the ventral neck midline. The digastric muscles were retracted to visualize the glossopharyngeal nerve passing between the carotid arteries and transected. The circumvallate papilla has bilateral innervation of the circumvallate papilla; hence, the nerve was transected bilaterally. Sham-operated mice received the same procedure with the exception of transection of the glossopharyngeal nerve.

### Lineage tracing

All the transgenic mouse lines used were described previously and were maintained on a C57BL/6 background. Cre-mediated recombination was induced in mice by intraperitoneal injection of tamoxifen (T5648, Sigma, and St. Louis, MO) at a dose of 100 mg/kg for 5 days. Surgical procedures for GLx were performed 2 weeks after tamoxifen injection. The mice were sacrificed at 2 weeks, 4 weeks, and 6 weeks after GLx, and their tongues were harvested.

### Histology and immunofluorescence

The samples were fixed in 4% paraformaldehyde and processed as per the standard procedure. Seven-micron-thick sections were prepared for hematoxylin/eosin staining and immunostaining. The specimens were boiled in citrate buffer (pH 6.0) for antigen retrieval and blocked using 1% goat serum or 5% bovine serum albumin in PBS. The specimens were incubated with primary antibodies against Gα gustducin (sc-395, Santa Cruz Biotechnology, Inc., USA, 1:100), SNAP25 (sc-20038, Santa Cruz Biotechnology, Inc., USA, 1:100), BrdU (ab6326, Abcam, UK, 1:200), IdU (MA5-24879, Invitrogen, USA, 1:200), c-Kit (ab231780, Abcam, UK; 1:100), PCNA (ab18197, Abcam, UK; 1:200), tdTomato (600-401-379, Rockland, PA, USA, 1:200), K14 (ab7800, Abcam, UK, 1:200), K8 (TROMA-I, DSHB, IA, USA, 1:200), Sox2 (AF2018, R&D, MN, USA, 1:40), Tuj1 (ab18207, Abcam, UK; 1:200) and Trpm5 (Guinea pig polyclonal anti-Trpm5 antibody was generated against amino acids residues 1089–1158 of Trpm5) at 4 °C overnight. The following day, these sections were incubated with a secondary antibody (1:200, Invitrogen, United States) and counterstained with TO-PRO^TM^-3 (T3605, Invitrogen, OR, USA; 1:1000) or DAPI (D1306, Invitrogen, OR, USA; 30 nM) to visualize nuclei. All specimens were examined using a confocal laser microscope (DMi8, Leica, Germany).

### Organoid culture

Tongues from sacrificed adult mice were dissected and injected with ~0.5 mL of Dispase II (4942078001, Roche, IN, USA) in PBS for 25 min at 37 °C. The tongue epithelium was gently detached from the underlying tongue mesenchyme. The epithelium of circumvallate and foliate papillae was dissected and incubated with TrypLE^TM^ (12604013, Gibco, Denmark) for 30 min at 37 °C and centrifuged at 800 rpm for 20 min. The cell pellet was resuspended in Matrigel (356234, Corning, NY, USA) and seeded onto 24-well culture plates (50 μL of Matrigel). Matrigel was allowed to polymerize for at least 10 min at 37 °C. Taste culture medium based on DMEM/F12 (11320033, Gibco, MA, USA) with N2 (17502048, Gibco, MA, USA), B27 (17504044, Gibco, MA, USA), R-spondin-1 (120-38, Peprotech, NJ, USA, 200 ng/mL), Noggin (120-10 C, NJ, USA, 100 ng/mL), Jagged-1 (188-204, Anaspec, CA, USA, 1 μM), Y27632 (1254, Tocris, Korea, 1 μM), N-acetylcysteine (A7250, Sigma, MO, USA 1 mM), and epidermal growth factor (AF-100-15, Peprotech, NJ, USA, 50 ng/mL) was added to the plate. Growth media were changed every 3 days.

### RT‒qPCR

The mouse circumvallate papilla epithelium was dissected out using Dispase II treatment, and the total RNA from the epithelium was extracted using TRIzol® reagent (#15596-026, Thermo Fisher Scientific, USA). The extracts were reverse transcribed using Maxime RT PreMix (#25081, iNtRON, Korea). RT‒qPCR was performed using a StepOnePlus Real-Time PCR System (Applied BioSystems, USA). RT–qPCR was performed using a StepOnePlus Real-Time PCR System (Applied Biosystems). The expression levels of each gene are expressed as normalized ratios against the B2m housekeeping gene.

*Lgr5*-F: 5'-AGC ATG CTT CTG GCA AGA TGT TC-3'; R: 5'-GAC TTA ACG CCC TGC GTT TGA-3', *K14*-F: 5'-AAT TCT CCT CAT CCT CTC AA-3'; R: 5'-CAT GTA GCA GCT TTA GTT CT-3', *Shh*-F: 5'-CGG ACC TTC AAG AGC CTT ACC-3'; R: 5'-GCA TAG CAG GAG AGG AAT GC-3', *Gli1*-F: 5'-TCA GCT GGA CTT TGT GGC TA-3'; R: 5'-GGC ACC TCA TGT AGC CAT TT-3'.

### Cell profile counting

In each section on a slide, immunostained cells were counted by manually counting visible cell profiles (elongated cells with a clear nucleus) in CVP along the lateral trench walls of the circumvallate papilla, and taste bud cells on the dorsum of the papilla were not included in the counting.

### Statistical analysis

The graphical results are expressed as the mean ± standard deviation (SD). GraphPad Prism 7 (GraphPad Software, San Diego, CA, USA) was used to analyze the data. Comparison of two groups was performed using an unpaired two-tailed *t*-test. Comparisons of multiple groups were performed by one-way ANOVA followed by Tukey’s multiple comparisons test. A *p-*value < 0.05 was considered significant.

## Results

### Taste buds contain label-retaining cells (LRCs)

**Fig. 1:**
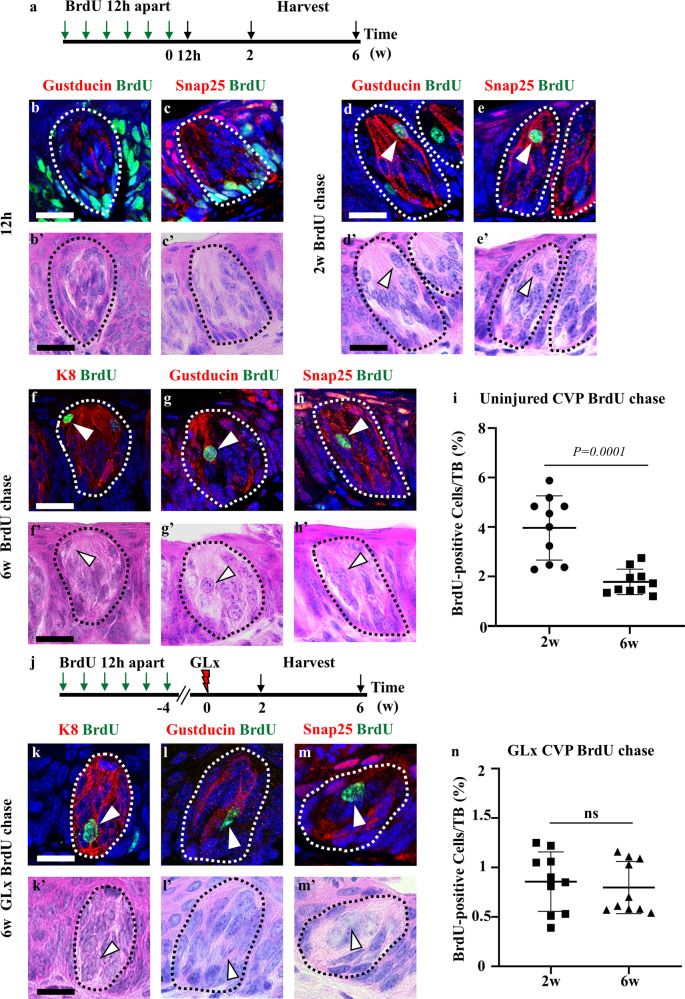
BrdU-positive label-retaining cells are maintained in CVP taste buds during homeostasis and regeneration. **a** Schematic of the experimental design for the identification of label-retaining cells. **b**, **c** At 12 h after the last injection (no chase), the proliferative basal cells are incorporated with BrdU label (green). **b'**, **c'** HE staining reveals the basal cells labeled with BrdU in the CVP epithelium. **d**, **e** BrdU-positive label-retaining cells (green) are observed within the taste buds after 2 weeks of the chase period. The label-retaining cells are colocalized with Type II and Type III taste receptor cells that are marked by gustducin and Snap25, respectively. **d'**–**e'** H&E staining of the taste buds of the CVP after 2 weeks of BrdU treatment. Arrowheads indicate BrdU-positive label-retaining cells expressing Type II or Type III taste receptor cell markers. **f** BrdU-positive label-retaining cells with pan-taste receptor marker K8 are observed in the taste buds after 6 weeks of the chase period. **g**, **h** The label-retaining cells are colocalized with Type II and Type III taste receptor cells. **f'**–**h'** H&E staining reveals taste buds of the CVP after 6 weeks of BrdU treatment. Arrowheads indicate BrdU-positive label-retaining taste receptor cells. **i** Quantification of BrdU-positive cells was performed in the CVP at 2 and 6 weeks after BrdU injection. Label-retaining cells were gradually reduced after BrdU injection, and a small number of cells were maintained at 6 weeks. **j** The schematic indicates the experimental procedure for the identification of label-retaining cells after GLx. Nerve transection was performed at 4 weeks after BrdU injection, and mice were sacrificed at 2 and 6 weeks after GLx. **k** A small number of BrdU-positive label-retaining cells are observed in the taste buds at 6 weeks after GLx. **l**, **m** The label-retaining cells are colocalized with Type II (gustducin) and Type III (Snap25) taste receptor cell markers. **k'**–**m'** H&E staining indicates the regenerated taste bud structure of the CVP at 6 weeks after GLx. The arrowhead indicates BrdU-positive taste receptor-retaining cells. **n** The number of BrdU-positive label-retaining cells in the circumvallate papilla was not significant among the groups at 2 and 6 weeks after GLx. *n* = 10 per condition. Data on the graph are displayed as the mean ± SD. Scale bar: 25 µm.

BrdU was pulsed daily for 6 days to label proliferating cells and determine the distribution of LRCs in the taste buds. Mice were sacrificed, and tissues were harvested at 12 h (no chase period) or 2 and 6 weeks (chase period) (Fig. 1a). Mice sacrificed after 12 h revealed that proliferating cells were enriched in the perigemmal region of the taste bud. Additionally, some of the newly differentiated cells or precursor cells could be observed inside the taste buds (Fig. 1b, c). The localization of BrdU-positive cells was further confirmed by HE staining (Fig. 1b', c’). BrdU label retention was used to identify slow-cycling cells in the taste buds of CVP. After 2 weeks of chase, Gα gustducin (Type II taste receptor cell marker)- and Snap25 (Type III taste receptor cell marker)-positive taste receptor cells exhibited BrdU-positive nuclei (Fig. 1d and e). We observed 4.17 ± 1.6 SD of taste receptor cells containing BrdU-positive nuclei (Fig. 1i), and these cells were localized inside the taste buds, as indicated by HE staining (Fig. 1d' and e'). Label retention at 6 weeks indicated that a subset of taste receptor cells, specifically Type II and Type III taste receptor cells, were LRCs (Fig. 1f–h), albeit with fewer BrdU-positive cells (1.77 ± 0.85 SD) (Fig. 1i). HE staining confirmed the localization of LRCs within the taste bud (Fig. 1f’–h’). The number of LRCs was significantly different between 2 and 6 weeks after chasing (Fig. 1i).

Transection of the glossopharyngeal nerve leads to taste bud degeneration. To quantify the number of *K8*-positive taste receptor cells that underwent degeneration and regeneration, we performed GLx, and the tongue was harvested at various time points (Supplementary Fig. 1a). The glossopharyngeal nerve was transected as shown in the surgical view (Supplementary Fig. 1b). The sham surgery mice possessed intact taste buds with nerves (Supplementary Fig. 1c and c’, arrowhead). Two weeks after GLx, a small number of *K8*-positive taste receptor cells were maintained in a manner that was independent of innervation in the CVP (Supplementary Fig. 1d, arrow). The taste bud structure was completely degenerated at 2 weeks after GLx, as revealed by HE staining (Supplementary Fig. 1d'). Six weeks after GLx, taste buds regenerated, and innervation was observed in the newly formed taste buds (Supplementary Fig. 1e, arrowhead). Regenerated taste bud structure was observed at 6 weeks after GLx compared to that at 2 weeks (Supplementary Fig. 1e'). Quantification of *K8*-positive taste receptor cells showed a significant reduction at 2 weeks and an increase at 6 weeks after GLx (Supplementary Fig. 1f). To identify the LRCs during regeneration, we injected BrdU, and GLx was performed on adult mice. The tongue tissues were collected at 2 and 6 weeks after GLx (Fig. 1j). Immunohistochemical staining revealed that a subset of K8-, gustducin-, and Snap25-positive taste receptor cells contained BrdU (Fig. 1k–m, arrowheads). This result indicated that the LRCs remained without degeneration in the absence of nerves and could be observed in the taste buds regenerated at 6 weeks. HE staining results indicated that BrdU-positive cells were detected in regenerated taste buds at 6 weeks after GLx (Fig. 1k’–m’ arrowheads). Quantification of BrdU-positive cells revealed that the number of BrdU-positive cells was similar between 2 and 6 weeks after GLx (Fig. 1n), thus indicating that the number of LRCs remained constant. No LRCs were observed outside the taste buds at 6 weeks in homeostasis and regeneration (Supplementary Fig. 1g and h). IdU was injected 1 h prior to sacrifice to investigate whether LRCs entered the cell cycle during regeneration. LRCs in taste buds colocalized with both BrdU and IdU, indicating that LRCs proliferate during regeneration (Supplementary Fig. 1i–m).

### *Lgr5-*negative stem/progenitor or *K14*-negative progenitor cells contribute to regeneration of taste receptor cells after nerve injury

**Fig. 2:**
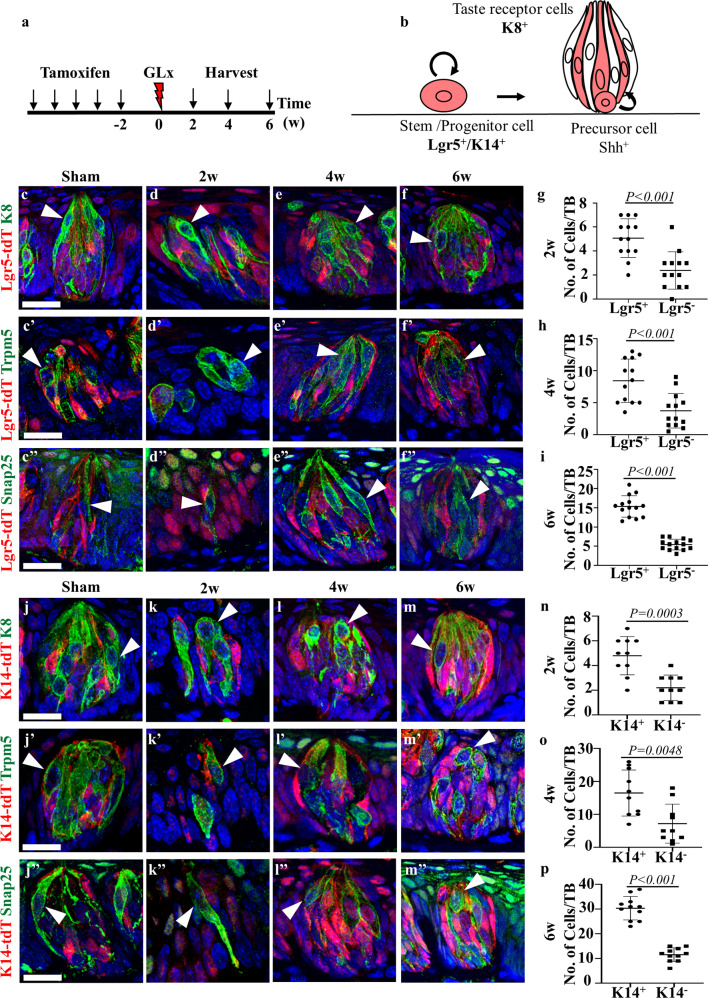
Heterogeneous cell populations contribute to taste bud regeneration after GLx. **a** Schematic of the experimental design for lineage tracing of *Lgr5*-positive stem/progenitor cells. **b** Schematic representation of the outcome of *Lgr5* stem/progenitor and *K14* progenitor cell lineage tracing. **c**–**c''** Taste buds from the sham surgery mice indicate that not all taste receptor cells are derived from *Lgr5*-positive stem/progenitor cells during homeostasis (arrowheads). **c** The taste buds are stained with the pan-taste cell marker K8, (**c'**) Type II taste receptor cell marker Trpm5, and (**c''**) Type III cell marker Snap25. **d** At 2 weeks after GLx, *K8*-positive taste receptor cells (green) remain in the CVP epithelium. Immunohistochemistry results revealed that the surviving taste receptor cells contain Type II and Type III taste receptor cells that are marked by (**d'**) Trpm5 and (**d''**) Snap25, respectively. **e**‒**e''** At 4 weeks after GLx, the taste bud structure was observed by regeneration. *Lgr5*-derived taste receptor cells are observed with tdTomato expression (red). A subset of regenerated taste receptor cells does not express tdTomato. Regenerating taste receptor cells stained with (**e**) K8, (**e'**) Trpm5, and (**e''**) Snap25 indicate that a subset of Type II and Type III taste receptor cells are not derived from *Lgr5*-positive taste stem/progenitor cells. **f**–**f''** At 6 weeks after GLx, tdTomato-negative and (**f**) K8-, (**f'**) Trpm5-, and (**f''**) Snap25-positive cells were detected in regenerating taste buds. **g** Quantification of surviving taste receptor cells at 2 weeks after GLx reveals that *K8*-positive, tdTomato-positive cells are more abundant than *K8*-positive and tdTomato-negative cells. **h**, **i** Quantification of the surviving taste receptor cells at 4 and 6 weeks after GLx reveals that the number of *K8*- and tdTomato-positive cells is increased compared to the number at 2 weeks after GLx; however, the number of *K8* cells not derived from *Lgr5* stem cells is similar at 2, 4 and 6 weeks after GLx. *K14* marks the progenitor cells in the circumvallate papilla that differentiate into taste receptor cells. **j**–**j''** Taste buds of the sham surgery mice indicate that not all taste receptor cells are derived from *K14*-positive progenitor cells during homeostasis (arrowheads). **j** The taste buds are stained with the pan-taste cell marker K8, (**j'**) the Type II taste receptor cell marker Trpm5, and (**j''**) the Type III cell marker Snap25. **k**–**k''** Similar to *Lgr5* stem/progenitor cell staining, a subset of taste receptor cells did not express the *K14*-derived tdTomato signal in the remaining taste receptor cells at 2 weeks after injury (arrowheads). **l**–**l''** During the initial phase of regeneration at 4 weeks after GLx, a subset of taste receptor cells did not arise from the *K14* progenitor cells (arrowheads). **m**–**m''** At 6 weeks after GLx, a subset of regenerated taste receptor cells is not derived from progenitor cells (arrowheads). **n**–**p** Quantification of surviving taste receptor cells at 2, 4 and 6 weeks after GLx reveals that the number of *K8* cells derived from *K14* is higher at all three time points. *n* = 10 per condition. Data on the graph are displayed as the mean ± SD. Scale bar: 25 µm.

*Lgr5*-positive stem cells are localized in the trench region of the CVP and give rise to new taste receptor cells involved in homeostasis and regeneration^8,9^. *Lgr5*^EGFP-CreERT2/+^; *R26R*^Tom/+^ and *K14*^CreERT/+^; *R26R*^Tom/+^ mice were pulsed with tamoxifen for 5 days, and the tissues were harvested at various time points (Fig. 2a). Lineage tracing of *Lgr5* stem/progenitor and *K14* progenitor cells was performed to determine whether all taste receptor cells were derived from these stem/progenitor cells during regeneration (Fig. 2b). *Lgr5*-derived tdTomato cells were observed in the taste buds of the sham surgery mice at 2 weeks, indicating that *Lgr5* cells give rise to taste and nontaste epithelium during homeostasis (Fig. 2c–c''). Interestingly, a subset of *K8*-positive taste receptor cells was not derived from *Lgr5* cells in the sham surgery mice, as indicated by the lack of tdTomato fluorescence, thus indicating that they might arise from different stem/progenitor cells (Fig. 2c–c'', arrowheads). Immunofluorescence staining of Trpm5 and Snap25 also revealed that a subset of Type II and III taste receptor cells, respectively, were not derived from *Lgr5* stem cells under homeostatic conditions (Fig. 2c' and c''). Immunofluorescence staining at 2 weeks after GLx revealed that a subset of the remaining taste receptor cells did not express *Lgr5*-derived tdTomato, thus indicating that *Lgr5*-negative cells may be derived from a different stem cell source, such as LRCs (Fig. 2d–d'' arrowheads). Four weeks after GLx, taste buds began to be regenerated, and taste bud structures were observed compared to observations at 2 weeks after GLx. *Lgr5*-derived tdTomato-positive cells were present in the regenerated taste buds, indicating that *Lgr5*-positive stem/progenitor cells are involved in the regeneration of taste buds (Fig. 2e–e''). Among the *Lgr5*-derived tdTomato-positive cells in regenerated taste buds, a subset of taste receptor cells did not express tdTomato (Fig. 2e–e'' arrowheads). Six weeks after GLx, the taste buds still contained taste receptor cells that did not express *Lgr5*-derived tdTomato (Fig. 2f–f'', arrowheads). Quantification of taste receptor cells at 2, 4 and 6 weeks after GLx revealed an increase in the number of *K8*-positive cells that were derived from *Lgr5-*positive stem cells; however, there was a subset of taste receptor cells not derived from *Lgr5*-positive cells in taste buds (Fig. 2g–i).

*K14*-positive cells are basally localized progenitor cells that renew and replenish taste receptor cells under homeostatic conditions^7^. *K14*-derived tdTomato-positive cells in the sham mice taste buds confirmed that *K14*-positive progenitor cells renew the taste receptor cells during homeostasis (Fig. 2j–j''). Similar to *Lgr5* lineage tracing, a subset of *K8*-positive taste receptor cells did not express *K14*-derived tdTomato (Fig. 2j arrowhead). Additionally, immunofluorescence targeting Trpm5 and Snap25 confirmed that the *K14* tdTomato-negative cells belonged to the Type II and Type III taste cells, respectively, in the sham group (Fig. 2j' and j'', arrowheads). Immunofluorescence performed at 2 weeks after GLx revealed that the taste bud structure was degenerated and that the remaining taste receptor cells did not express *K14*-derived tdTomato, similar to *Lgr5* lineage tracing (Fig. 2k–k'' arrowheads). The regenerating taste buds after 4 weeks of GLx exhibited a subset of taste receptor cells that did not express *K14*-derived tdTomato belonging to both Type II and Type III taste receptor cells (Fig. 2l–l'' arrowheads). Six weeks after GLx, a subset of taste receptor cells did not express *K14*-derived tdTomato, similar to previous observations (Fig. 2m–m'' arrowheads). Quantification of taste receptor cells at 2, 4, and 6 weeks after GLx showed an increase in the number of *K8*-positive cells derived from *K14*; however, there was a population of taste receptor cells that were not *K14* derived (Fig. n–p). These results indicate the existence of a cell pool within the taste bud that is independent of *Lgr5*-positive stem/progenitor or *K14*-positive progenitor cells.

### *K14* progenitor cells exhibit elevated gene expression and costaining with taste receptor cells in nerve-transected mice

**Fig. 3:**
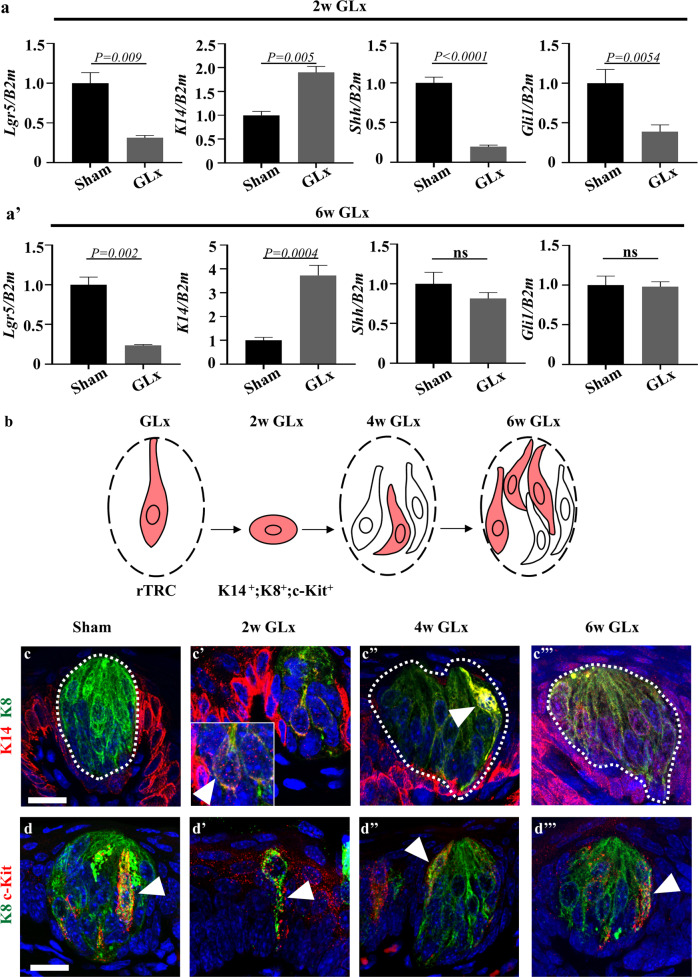
Mature taste receptor cells participate in taste bud regeneration by acquiring progenitor cell-like characteristics after injury. **a** The expression levels of *Lgr5*, *Shh*, and *Gli1* were decreased at 2 weeks after GLx compared to the levels in the sham mice. The *K14* expression level was significantly higher after GLx treatment than that in the sham mice. **a'** *Lgr5* expression is reduced after GLx compared to that in the sham group. *K14* expression is higher in GLx mice than in sham mice*. Shh* and *Gli1* expression levels were not significantly different between the sham and GLx groups. **b** Schematic of the dedifferentiation pathway. **c**–**c'''** Immunofluorescence staining was performed using the progenitor cell marker K14 and the mature taste receptor cell marker K8. **c** *K8*-positive cells are localized within the taste bud, and K14-positive cells are observed in the perigemmal region outside of the taste bud. **c'** At 2 weeks after GLx, only a small number of cells remain that exhibit colocalization of K8 and K14 (arrowhead). **c''** At 4 weeks after GLx, a subset of cells within the regenerating taste bud exhibit K8 and K14 colocalization (arrowhead). **c'''** At 6 weeks after GLx, a small number of regenerating taste receptor cells exhibit K8 and K14 costaining (arrowhead). **d** c-Kit expression is observed in a subset of taste receptor cells in the sham surgery mice. **d'** c-Kit is expressed in the remaining cells at 2 weeks after GLx. **d''** and **d'''** Regenerated taste receptor cells after GLx also exhibit expression of c-Kit (arrowhead). rTRC remaining taste receptor cells, *n* = 10 per condition, Scale bar: 25 µm.

Real-time quantitative polymerase chain reaction (RT‒qPCR) of stem, progenitor, and precursor cell genes was performed to evaluate changes in gene expression. Two different time points (2 and 6 weeks) after GLx were selected to identify gene expression levels in the degeneration and regeneration phases, respectively. The stem cell gene *Lgr5* was unaltered after GLx compared to that in the sham group at both examined time points. However, the progenitor cell gene *K14* was significantly upregulated after GLx in the CVP epithelium (Fig. 3a and a'). *Shh* is predominantly expressed in taste precursor cells^24^, and its responsive gene *Gli1* was downregulated at 2 weeks after GLx, while the expression levels returned to normal at 6 weeks during regeneration (Fig. 3a and a'). The schematic diagram indicates the dedifferentiation of the remaining taste receptor cells into *K14*-positive progenitor cells to regenerate taste buds (Fig. 3b). Immunofluorescence staining of K14 and K8, which mark progenitor cells and taste receptor cells, respectively, was performed to investigate the *K8*-positive taste receptor cell transition into *K14* progenitor cells. In the sham group, *K14*-positive progenitor cells were localized within the perigemmal region of the taste bud, and *K8*-positive taste receptor cells were localized intragemmally (Fig. 3c). At 2 weeks after GLx, a small number of K8 and K14 colocalized cells were detected in degenerating taste buds (Fig. 3c'). At 4 and 6 weeks after GLx, *K14*-positive progenitor cells were observed inside the taste buds and colocalized with *K8* (Fig. 3c'' and c'''). c-Kit is a tyrosine kinase receptor, and activation of c-Kit with its associated pathway protects cells from apoptosis^25^. To identify whether c-Kit is involved in the survival of taste receptor cells, we performed immunohistochemistry of CVP after GLx. c-Kit was observed in a subset of taste receptor cells in the CVP after sham surgery (Fig. 3d). c-Kit was localized in the remaining taste receptor cells at 2 weeks after GLx (Fig. 3d'). c-Kit-positive cells were also observed in the subset of taste receptor cells at 4 and 6 weeks after GLx (Fig. 3d'' and d''').

### *K8*-derived dedifferentiated cells give rise to taste receptor cells and nontaste epithelium during regeneration

**Fig. 4:**
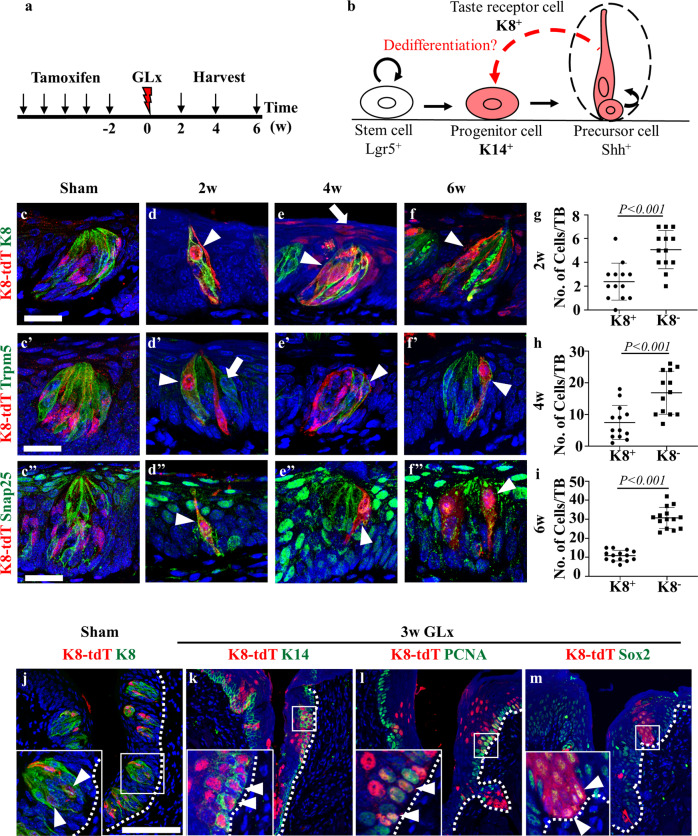
The mature taste receptor expressing *K8* dedifferentiates into progenitor-like cells expressing *K14* after GLx. **a** Schematic of the experimental procedure for lineage tracing of *K8*-positive taste receptor cells. **b** Schematic of lineage labeling of the taste receptor cells and dedifferentiation after GLx. Tamoxifen-induced Cre activates tdTomato fluorescent protein (red), which labels *K8* cells and their progeny in taste buds. **c**–**c''** A subset of taste receptor cells in the sham surgery mice expressed the tdTomato signal. **d**–**d''** Two subtypes of remaining taste receptors are observed at 2 weeks after GLx, the remaining taste receptor cells (arrowhead) expressing tdTomato and newly formed taste receptor cells (arrow) derived from stem/progenitor cells during the chase period. **d'** The remaining taste receptor cells express Trpm5 and (**d''**) Snap25, thus indicating the presence of Type II and Type III taste receptor cells. **e**‒**e''** At 4 weeks after GLx, *K8*-derived taste receptor cells are observed in the taste bud (arrowhead) and nontaste epithelial cells (arrow). **e'** Trpm5-expressing (Type II) and (**e''**) Snap25-expressing (Type III) taste receptor cells are derived from the remaining *K8*-positive cells (arrowhead). **f** At 6 weeks after GLx, the regenerated taste receptor cells exhibit the presence of K8 (arrowhead). **f'** A subset of Trpm5-expressing (Type II) and (**f''**) Snap25-expressing (Type III) taste receptor cells are K8-positive at 6 weeks after GLx. **g** Quantification of surviving taste receptor cells at 2 weeks after GLx reveals that *K8*-positive, tdTomato-negative cells are more abundant than *K8*-positive and tdTomato-negative cells. **h**, **i** Quantification of the surviving taste receptor cells at 4 and 6 weeks after GLx reveals that the number of *K8*- and tdTomato-negative cells is increased compared to the number at 2 weeks after GLx; however, the number of *K8* cells derived from *K8*–positive differentiated cells is similar at 4 and 6 weeks after GLx. **j** *K8*-derived tdTomato-positive cells localized within the taste buds in the sham mice. **k**–**m** Three weeks after GLx, *K8*-derived cells were observed in the majority of the trench region of the CVP. The *K8-*derived cells express (**k**) the basal/progenitor cell marker K14, (**l**) proliferative cell marker PCNA, and (**m**) the taste progenitor marker Sox2. Data on the graph are displayed as the mean ± SD. Scale bar: **c**–**f''**—25 µm, **j**–**m**—100 µm.

To evaluate the role of the remaining taste receptor cells in the regeneration of taste buds, we performed lineage tracing in *K8*^CreERT2/+^; *R26R*^Tom/+^ mice to label the taste receptor and its derived cells. Tamoxifen was injected into the mice to activate Cre, and the mice were sacrificed at different time points after GLx (Fig. 4a). A schematic representation of the dedifferentiation of fully differentiated taste receptor cells into progenitor cells is shown (Fig. 4b). Two weeks after sham surgery, tdTomato-positive taste receptor cells were detected in taste buds (Fig. 4c–c''). At 2 weeks after GLx, two types of taste receptor cells remained. The tdTomato-expressing taste receptor cells were indicative of the remaining taste receptor cells (Fig. 4d, arrowhead) and newly formed taste receptor cells from stem/progenitor cells that did not express *K8*-derived tdTomato (Fig. 4d', arrow). Both Type II and Type III taste receptor cells remained 2 weeks after GLx, and these cells expressed *K8*-derived tdTomato (Fig. 4d' and d'', arrowhead). Four weeks after GLx, *K8*-derived tdTomato-positive cells were observed in the regenerating taste bud (Fig. 4e, arrowhead) and in the nontaste epithelium (Fig. 4e, arrow). *K8*-derived cells expressed tdTomato and colocalized with the Type II (Trpm5) and Type III (Snap25) taste receptor cell markers during regeneration at 4 weeks (Fig. 4e' and e'' arrowheads). Six weeks after GLx, *K8*-derived tdTomato-positive cells were still observed in the regenerated taste bud (Fig. 4f–f'', arrowhead). Quantification of *K8*-derived cells revealed that at 2, 4, and 6 weeks after GLx, a small number of *K8*-derived tdTomato-positive cells remained in the taste bud compared to *K8* tdTomato-negative cells (Fig. 4g–i). These results indicate that not only stem/progenitor cells but also remaining taste receptor cells after GLx participate in the regeneration of taste buds.

To further examine the regeneration of taste receptor cells by the remaining differentiated taste receptor cells, we sacrificed *K8*^CreERT2/+^; *R26R*^Tom/+^ mice at 3 weeks after GLx. In the sham surgery mice, the *K8* tdTomato signal was restricted to the taste buds (Fig. 4j). Three weeks after GLx, *K8* tdTomato-positive cells were observed in the CVP epithelium without the taste bud structure (Fig. 4k–m). A subset of *K8* tdTomato-positive cells was basally localized and colocalized with the progenitor cell marker K14 (Fig. 4k, arrowheads), and they were proliferative, as confirmed by the colocalization of the cell cycle protein PCNA (Fig. 4l, arrowheads). *K8* tdTomato-positive cells also colocalized with the lingual progenitor cell marker Sox2 at 3 weeks after GLx (Fig. 4m, arrowheads). These results indicated that the remaining taste receptor cells acquire stemness through cell plasticity after GLx and participate in the regeneration of the taste bud.

### *K8*-derived cells exhibit organoid formation in vitro

**Fig. 5:**
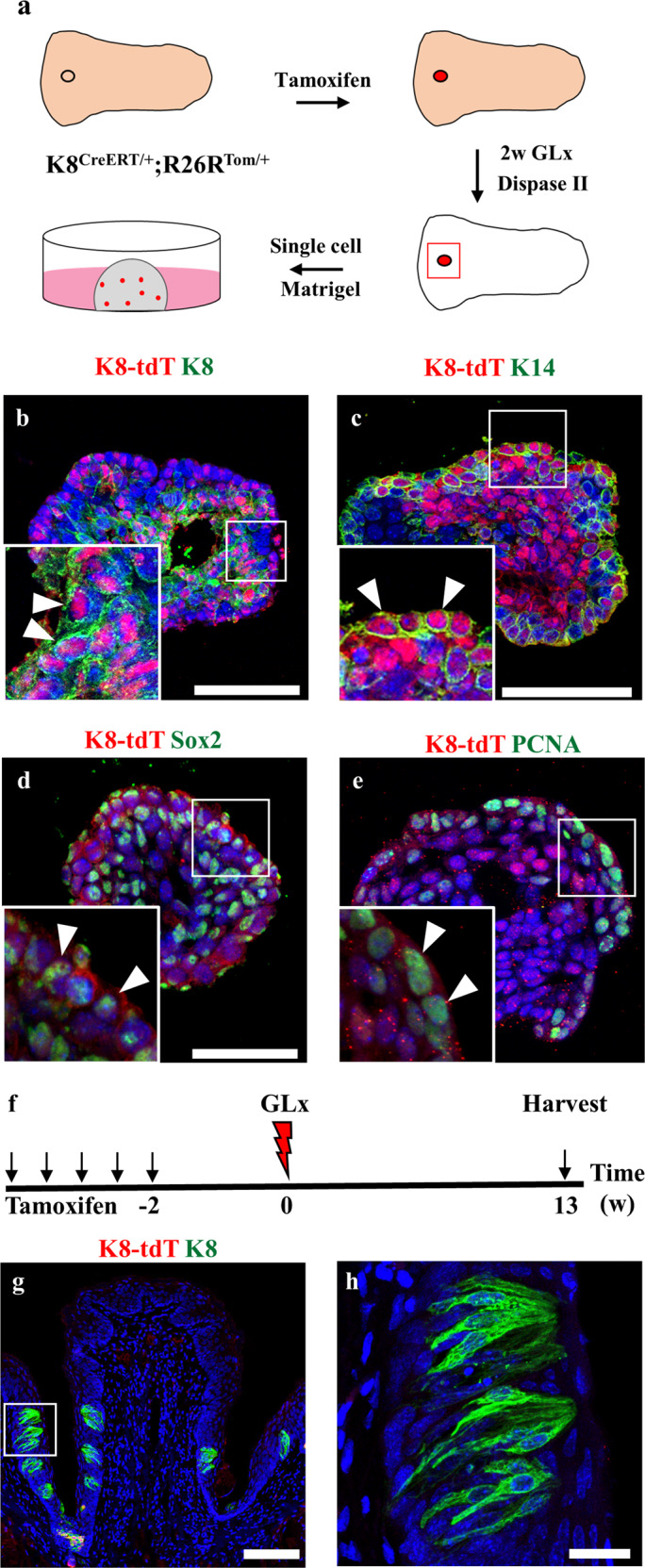
The remaining dedifferentiated taste receptor cells after GLx form organoids in vitro. **a** Schematic representation of the experimental procedure for the organoid formation assay. **b** *K8*-derived organoids contain mature taste receptor cells indicated by K8 and (**c**) basal/progenitor cells along the periphery of the organoid as marked by K14. Progenitor cells and proliferative cells are marked by (**d**) Sox2 and (**e**) PCNA, respectively, in organoids derived from *K8* taste receptor cells after injury. **f** Schematic of long-term (13 weeks) lineage labeling of *K8* cells after GLx. **g** *K8*-labeled cells were not observed in CVP at 13 weeks of chasing. **h** Higher magnification of the boxed area in **g**. Scale bar: **b**–**e**, **g**-100 µm, **h**-20 µm.

To examine whether these dedifferentiated cells possessed the capacity to form organoids in vitro, we cultured the CVP cells from *K8*^CreERT2/+^; *R26R*^Tom/+^ mice 2 weeks after GLx in Matrigel (Fig. 5a). After 14 days of Matrigel culture, the dedifferentiated *K8* tdTomato-positive cells formed organoids in vitro. The organoids formed from the remaining taste receptor cells expressed tdTomato fluorescence. *K8* tdTomato-positive organoids possessed differentiated taste receptor cells that were marked by K8, progenitor cells marked by K14 and Sox2 and proliferative cells marked by PCNA (Fig. 5b–e). These results indicated that the remaining differentiated taste receptor cells acquired the capacity to form taste bud organoids in vitro. Long-term lineage tracing of dedifferentiated *K8* tdTomato-positive cells revealed that the remaining taste receptor cells were short-lived and that they could be observed during the early phase of taste bud regeneration. *K8*-derived tdTomato-positive cells were not observed in taste buds after 13 weeks of long-term lineage tracing (Fig. 5f–h). These data indicate that the remaining *K8* tdTomato-positive cells dedifferentiate into transient progenitor cells to regenerate taste buds during regeneration.

## Discussion

**Fig. 6:**
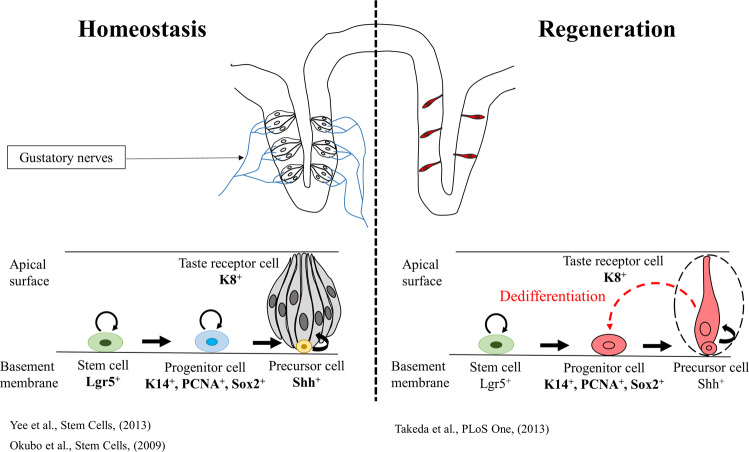
Dedifferentiation and stem cells drive regeneration of taste receptor cells. In homeostasis, renewal of the taste receptor cells is derived from *Lgr5*-positive stem cells in the circumvallate papilla. *Lgr5*-positive stem cells produce progenitor cells that express *K14, PCNA*, and *Sox2*. These progenitor cells further differentiate into postmitotic *Shh*-expressing precursor cells that finally transition into terminally differentiated taste receptor cells. Nerve transection leads to degeneration of the taste buds; however, a small number of taste receptor cells remain independent of innervation. The remaining taste receptor cells acquire stemness by dedifferentiating into transient progenitor-like cells expressing K14, Sox2, and PCNA, and they participate in the regeneration of taste buds along with *Lgr5*-positive stem/progenitor cells for rapid regeneration of the taste buds.

Taste receptor cells are sensory cells that employ an efficient mechanism for robust regeneration of taste receptor cells to maintain their function. In this study, we used a severe but reversible model of injury to investigate epithelial plasticity in taste bud regeneration. This study revealed that injury induced differentiated taste receptor cells to acquire progenitor cell-like characteristics and participate in the regeneration of the taste bud (Fig. 6).

LRCs represent a pool of stem/progenitor cells that are activated upon injury to regenerate the damaged tissue but remain quiescent during homeostasis, as demonstrated in teeth and salivary glands^26–28^. Our study demonstrated the presence of LRCs in taste buds that were maintained after injury and were observed in the regenerated taste buds (Fig. 1k–m). These results indicated that LRCs present in taste buds may represent a pool of facultative stem cells that are activated upon injury to regenerate the taste bud.

*Lgr5*-positive cells form new taste receptors and nontaste epithelial cells during homeostasis and regeneration^8,9^. Our lineage tracing data revealed that not all regenerated taste receptor cells were derived from *Lgr5*-positive stem cells. A subset of taste receptor cells was derived from *Lgr5*-negative cell populations, as indicated by the absence of *Lgr5*-derived tdTomato fluorescence (Fig. 2c–f''). Similar results were shown in other tissues, such as hair, colon and small intestine, in which *Lgr5*-positive stem cells are dispensable and regeneration can be achieved by other cells that acquire stemness by dedifferentiation^18,19,29^. Lineage tracing of progenitor cells (*K14*-positive) after GLx revealed that a subset of taste receptor cells did not express *K14-*derived tdTomato (Fig. 2j–m''). These data indicate that a population of stem/progenitor cells participated in the regeneration of taste receptor cells independent of *Lgr5*-positive stem and *K14*-positive progenitor cells. However, the possibility of incomplete penetrance of Cre recombination cannot be completely ruled out, resulting in the presence of K8-positive/Lgr5-tdT-negative cells.

In taste buds, basal cells are dependent upon nerve-derived *Shh* for their differentiation into taste receptor cells^30–32^. RT‒qPCR data revealed that at 2 weeks after GLx, the expression levels of *Shh* and its pathway-associated transcription factor *Gli1* were decreased. However, after 6 weeks of taste bud regeneration following reinnervation, the expression levels of *Shh* and *Gli1* were similar to those in the sham surgery mice, indicating that nerve-derived *Shh* was restored at 6 weeks after GLx.

*K14*, the stratified epithelial cell maker, is expressed in progenitor cells, and *K8*, the columnar epithelial cell maker, is expressed in fully differentiated taste receptor cells. Previously, these markers have been used as indicators, as they are expressed at different differentiation levels^33^. Keratin transition has been reported in tissues during development and differentiation, such as prostrate, esophagus and hair follicles^34–36^. Immunofluorescence results revealed that after injury, the differentiated taste receptor cells expressing *K8* and progenitor cell markers expressing *K14* colocalized within the same cell, which was not observed in the sham mice (Fig. 3c-c'''). These data indicate that the remaining *K8*-expressing taste receptor cells have undergone a transition into an immature cell state as *K14*-positive progenitor-like cells following GLx.

*K8*-positive taste receptor cells are fully differentiated cells that are postmitotic and are present exclusively within the taste bud. Lineage tracing of *K8*-positive taste receptor cells at different time points after injury demonstrated that *K8*-derived cells regenerate taste receptor cells and nontaste epithelial cells during the regeneration phase (Fig. 4c–f''). *K8-*derived cells were present in the basal region of the CVP epithelium and colocalized with K14, PCNA, and Sox2, indicating that the injury induced cellular plasticity in the differentiated taste receptor cells (Fig. 4k–m). Organoid formation is a characteristic of stem/progenitor cells, and differentiated cells cannot form organoids^37,38^. To confirm that the differentiated cells acquired stemness by undergoing dedifferentiation, we performed a taste bud organoid formation assay as described in our previous study^39^. Dedifferentiation has been observed in various tissues during regeneration; however, subtle differences in the mechanism of dedifferentiation are observed between the taste bud and other tissues. In the tracheal epithelium and intestine, dedifferentiation is observed following the ablation or loss of stem cells after injury. Differentiated cells were shown to dedifferentiate into stem/progenitor-like cells^40,41^. However, our study revealed that dedifferentiation can occur without the ablation or loss of stem cells and that the differentiated cells dedifferentiate into progenitor cells that do not persist for long-term (Fig. 5g, h).

c-Kit and its associated pathway protect cells from apoptosis and are also involved in cell plasticity^25,42^. c-Kit was localized in the subset of taste receptor cells in the sham surgery mice as well as in regenerating taste receptor cells after GLx (Fig. 3d–d''') and may be involved in the survival and plasticity of taste receptor cells after injury. The potential of c-Kit for the regeneration of taste receptor cells requires further investigation.

In the airway epithelium, basal stem cells prevent the dedifferentiation of secretory cells; however, after ablation of basal stem cells, secretory cells can dedifferentiate into basal stem cells^40^. Similarly, after nerve injury, Schwann cells lose contact with the axon they are myelinating and dedifferentiate into immature Schwann cells, mediated by the Ras/Raf/ERK signaling pathway^43,44^. In taste buds, the nerve or its derived factors may prevent dedifferentiation of taste receptor cells in homeostasis; however, after injury, this factor is lost, facilitating the dedifferentiation of surviving taste receptor cells. Further studies are necessary to better understand the pathways and microenvironmental signals that promote the cellular plasticity of taste receptor cells.

In summary, this study revealed that a subset of fully differentiated taste receptor cells survives after injury and undergo dedifferentiation and proliferation during regeneration. Our results revealed that both stem/progenitor and differentiated remaining taste receptor cells contribute to the regeneration of taste buds following GLx. Further studies are necessary to better understand the mechanism, pathways and microenvironmental signals that promote the cellular plasticity of taste receptor cells, which may aid in recovering the sense of taste lost due to COVID-19^45^. Additionally, *Lgr5*-DTR mice that possess the diphtheria toxin receptor gene in the *Lgr5* locus could be utilized to ablate *Lgr5-*positive cells and examine regeneration in the absence of stem/progenitor cells, as previously demonstrated in the intestine^46^. Single-cell RNA sequencing is a powerful tool that provides insights into the functions of specific cells. However, due to relatively few cells in the CVP of the mice, single-cell RNA sequencing could not be performed; thus, many questions remain unanswered that could be addressed in future studies^47^.

## Supplementary information

Supplementary Data
