# Supplementary material for: Epithelial plasticity enhances regeneration of committed taste receptor cells following nerve injury
Source: Exp Mol Med. 2023 Jan 11;55(1):171–82. doi: 10.1038/s12276-022-00924-8 (PMC9833027; doi:10.1038/s12276-022-00924-8)
Supplement: Supplementary file 1 — Supplementary Data [file 12276_2022_924_MOESM1_ESM.pdf]

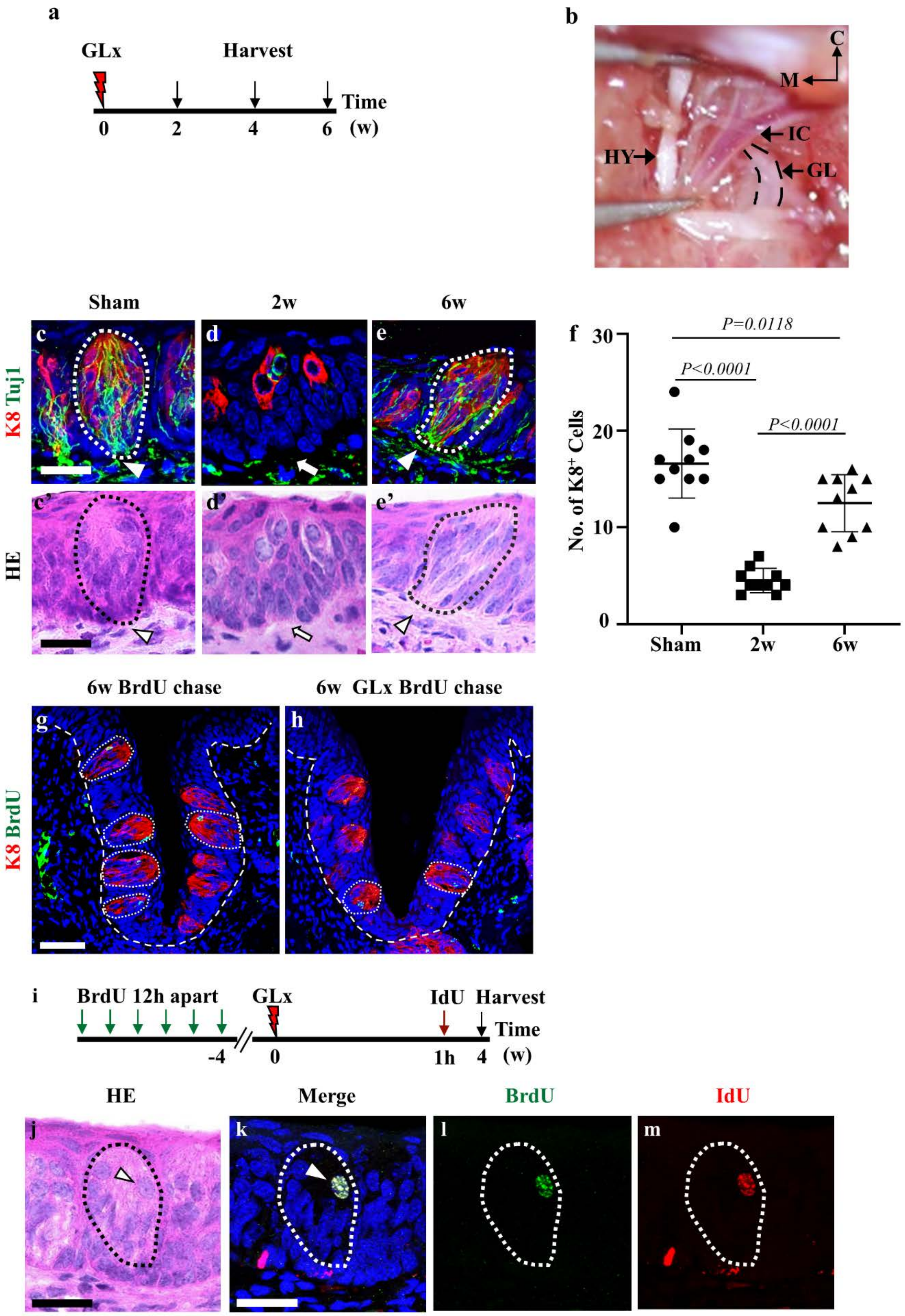

a
GLx
Harvest
Time
0
2
4
6
(w)
b
C
M
IC
HY
GL
Sham
2w
6w
c
d
e
K8 Tuj1
c'
d'
e'
HE
f
No. of K8⁺ Cells
P=0.0118
P<0.0001
P<0.0001
30
20
10
0
Sham
2w
6w
6w BrdU chase
6w GLx BrdU chase
g
h
K8 BrdU
i
BrdU 12h apart
GLx
IdU
Harvest
Time
-4
0
1h
4
(w)
HE
Merge
BrdU
IdU
j
k
l
m

**Supplementary Fig 1. A subset of taste cells remains independent of innervation**

(a) Schematics represent the Glossopharyngeal nerve transection and tissue harvest after GLx. (b) Picture of the surgical field demonstrating the exposure of the Glossopharyngeal nerve prior to transection. (c) Tuj1-positive nerve fibers are connected to *K8*-positive taste receptor cells in sham mice, and arrowheads indicate nerve fibers innervating the taste bud. (d) At 2 weeks after GLx, small number of taste receptor cells remain without nerve innervation. Arrow indicates the absence of nerve fibers innervating the epithelium. (e) At 6 weeks after GLx, taste buds are regenerated, and nerve fibers are innervated into CVP taste buds. Arrowhead indicates nerve fibers innervating the taste buds. (c'-e') H&E staining indicates that taste bud morphology is disrupted at 2 weeks after GLx, and at 6 weeks after GLx, taste buds are regenerated and exhibit similar structures compared to those of the sham group. (f) The number of *K8* taste receptor cells is significantly reduced at 2 weeks after GLx but increases at 6 weeks. (g-h) LRC's were observed only within the taste bud at 6 weeks in homeostasis and regeneration. (i) Schematics of the BrdU and IdU injection to identify proliferation of LRC's in response to injury. (j) HE staining indicating the proliferating LRC within the taste bud. (k-m) In taste bud, BrdU-positive LRC incorporated IdU during regeneration at 4 weeks after GLx. HY-Hypoglossal nerve, IC-Internal carotid artery, GL-Glossopharyngeal nerve. n=10. Data on graph are displayed as mean ± SD. Scale bar c-e', j-m: 25μm, g,h: 50μm
